# Out of the Niche: A Bird’s-Eye View of the Molecular Networks Controlling Root Stem Cells

**DOI:** 10.3390/plants14162574

**Published:** 2025-08-19

**Authors:** Giovanna Sessa, Giorgio Morelli, Massimiliano Sassi

**Affiliations:** 1 Istituto di Biologia e Patologia Molecolari, Consiglio Nazionale delle Ricerche (IBPM-CNR), 00185 Rome, Italy; giovanna.sessa@cnr.it; 2 Accademia Nazionale dei Lincei, 00165 Rome, Italy; giorgio.morelli.crea@gmail.com

**Keywords:** stem cell niche, quiescent center, distal stem cell, root apical meristem, hormones, transcription factors, differentiation, proliferation

## Abstract

The capacity of plants to generate new organs and tissues throughout their life cycle depends on the activity of the stem cells contained in meristematic tissues. Plant stem cells are organized in small, clustered populations referred to as stem cell niches. In addition to generating new undifferentiated cells, stem cell niches also provide the positional information that maintains stem cell self-renewal properties and controls the non-cell-autonomous differentiation of surrounding tissues. In this review, we aim to analyze and discuss the most recent literature describing the molecular mechanism controlling the activity and the organization of the stem cell niche in the root of the model plant *Arabidopsis thaliana* (L.) Heynh. In particular, we will focus on the complex molecular regulatory networks that control the balance between stemness and differentiation in distal stem cells, as well as the maintenance of the mitotically inactive state of the quiescent center.

## 1. Introduction

Unlike animals, which establish their body plan during embryogenesis, plants are characterized by indeterminate growth, i.e., new organs develop post-embryonically in a process that continues throughout the plant’s lifespan. Plant indeterminate growth depends on the activity of meristems, reservoirs of pluripotent cells localized at the tips of shoot and root growth axes that continuously produce new tissues [1,2]. In addition to the shoot and root apical meristems (hereinafter SAM and RAM, respectively), plants also possess a cambial meristem surrounding the vasculature that regulates radial growth [3]. Moreover, meristems can be formed de novo from mature cells to generate new growth axes, as in the case of lateral root primordia and lateral branches, or in response to wounding to regenerate damaged parts [4]. Regardless of the meristem type, pluripotent meristematic cells derive from a small group of stem cells, also called initials, embedded within the tissue. The initials are always associated with an adjoining set of mitotically quiescent cells that constitute the organizing center which, by acting non-cell-autonomously, confers stemness to the abutting initials [1,3,5]. The initials and organizing center are collectively known as the stem cell niche (SCN), a cellular microenvironment of crucial importance for the growth of plants, as it coordinates the activity of the meristem in which it is contained. The importance of the SCN is further highlighted by the fact that, upon surgical or laser-mediated ablation, a new SCN can be regenerated by meristematic cells, both in shoot and root tissues, to ensure proper continuity in plant developmental plans [6,7,8]. Thus, SCNs are crucial to plants’ growth and development and understanding their functions is key to plant biology research and crop improvement.

In this review, we will discuss the SCN of the root apical meristem and we will describe the molecular networks regulating its maintenance and the control of stemness. In this regard, we will specifically focus on a subset of root stem cells—the distal stem cells (DSCs)—and we will highlight its interdependence with the SCN organizer—the quiescent center (QC)—in regulating the balance between stemness and differentiation.

## 2. Structure and Function of the Root Stem Cell Niche

**Figure 1:**
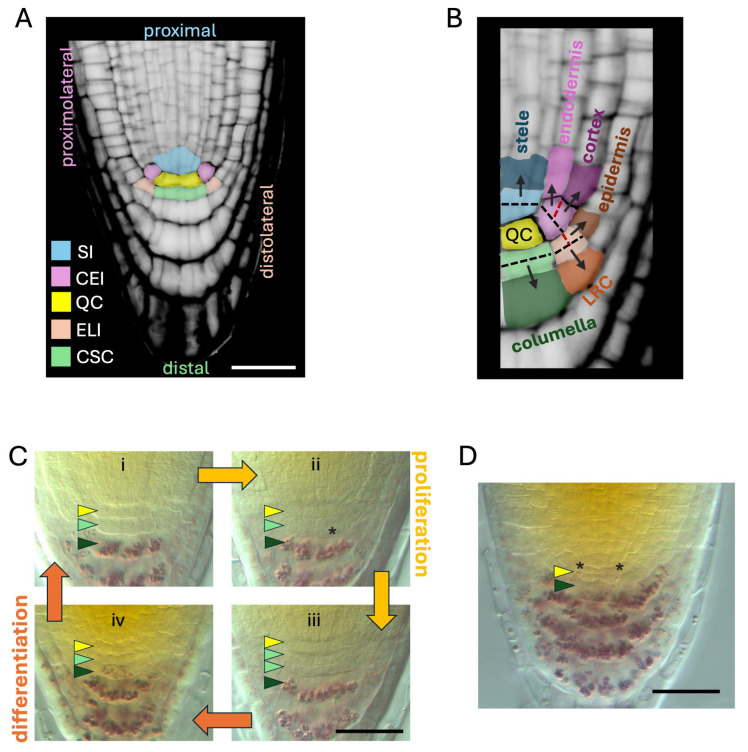
The SCN in *Arabidopsis thaliana*. (**A**) Structure of the SCN, depicted by overlying cell type-specific colors over a Confocal Laser Scanning Microscopy image depicting a propidium iodide-stained meristem from a 5-day-old wild-type root. SI, stele initials; CEI, cortex/endodermis initials; QC, quiescent center; ELI, epidermis/lateral root cap initials; CSC; columella stem cells. (**B**) Graphical representation of formative divisions and lineages of the root stem cells. Initials are color coded as in (**A**); daughter cells are depicted in darker tones compared to their respective initials. Dashed lines represent the orientation of cell division planes for each initial. Black dashes, first division plane; red dashes, second division plane in CEI and ELI. The image is a higher magnification detail of (**A**). (**C**) Cycles of proliferative (yellow arrows) and differentiative (orange arrows) phases in CSCs displayed in 5-day-old wild-type roots stained with Lugol’s solution and imaged under Nomarski optics upon chloral hydrate clearing. (i) A single layer of CSCs (light green arrowhead) is contained between the QC (yellow arrowhead) and differentiated columella cells (dark green arrowhead); (ii) CSCs start dividing to give rise to CSCDs (asterisk); (iii) when all the columella initials have divided, the formation of a double-layered structure of CSC-like cells can be observed; (iv) CSCDs start differentiating, ultimately restoring the conformation observed in (i). (**D**) The QC acts as a reservoir of stem cells in cases of extreme differentiation of CSCs. The image shows an RAM where CSC differentiation was induced by altering local auxin gradients with naphtylphtalamic acid treatments (10 μM, 5 days). Notice QC divisions (asterisks) adjacent to differentiated columella cells marked by purple staining of starch granules using Lugol’s solution. Scale bars, 20 μm.

In most plant species, the root SCN is located at the very tip of the organ and it is isolated from the environment by a protective layer, the root cap [9,10]. In the model plant *Arabidopsis thaliana*, the SCN can be easily identified in optical or histological longitudinal sections of roots by its relatively simple and invariant structure (Figure 1A) [11,12]. The SCN consists of a small group of cells with low mitotic activity, the QC, surrounded by a set of stem cells, which are the origin of all the tissues in the root. The initials located proximally (i.e., facing the shoot) or laterally to the QC generate the tissues that compose the radial structure of the root: the vasculature, the pericycle, the endodermis, and the cortex (Figure 1A,B). These initials undergo asymmetric cell division to generate daughters that will form a transit-amplifying population of pluripotent stem cells before differentiating. These transit-amplifying cells constitute the proximal RAM, and their proliferative activity is required for root elongation [13,14]. On the other hand, the stem cells placed distally to the QC are characterized by different behaviors. The initials placed distolaterally to the QC alternate the orientation of formative cell division planes to give rise to the two outermost tissues of the mature root (Figure 1A,B): the first asymmetric cell division occurs periclinally and generates the lateral root cap (LRC); the second division is oriented anticlinally to generate the epidermis [15]. The epidermal cells will undergo transit-amplifying divisions following the cells of adjacent cortex, whereas LRC cells differentiate after a few rounds of divisions [10]. Finally, the stem cells placed directly below the QC—the columella stem cells (CSCs)—display the simplest behavior among root initials. In fact, CSCs undergo a single periclinal division which generates two daughters with distinct fates: the apical daughter (i.e., the one adjoining the QC) retains the stem cell identity, whereas the daughter farthest from the QC (also known as columella stem cell daughter, CSCD) will soon differentiate into a mature columella cell by accumulating starch granules in the amyloplasts for gravity-sensing (Figure 1C) [1,10]. The columella, together with the LRC, form the root cap, a protective layer which is constantly sloughed off during growth, releasing secretory cells that facilitate root penetration into the soil [10,15]. Because of the continuous shedding, the division rates of CSC and LRC initials must be tightly coupled and synchronized to maintain a constant size of the root cap [12]. An invariant columella root cap size is also crucial to keep the SCN in a fixed position within the RAM, ensuring proper meristematic functionality [1,15].

In recent decades, the arabidopsis root SCN has clearly become a model system to study stem cells in plants. CSCs offer a particularly simple and convenient system to investigate the factors that control the balance between stemness and differentiation. From this perspective, CSCs also offer an ideal platform to analyze the role of the QC as an SCN organizer, since the interdependence between these stem cell compartments is well-established [16,17,18].

## 3. The QC-CSC Interdependence

In arabidopsis, the QC is composed of 4–8 brick-shaped cells with very poor mitotic activity compared with the high proliferation rates of the surrounding initials [11,19,20]. In young plants, QC cells might take up to 7 days to enter into the S phase [18], although their division frequencies tend to increase in older roots [19,21,22]. Because of their low mitotic activity, QC cells are believed to be less prone to DNA damage, and thereby act as a long-term reservoir of stem cells for the entire SCN [9]. Indeed, fast-proliferating initials are damaged by genotoxic stress and die within a few hours, but they are quickly replaced by QC daughter cells once the stress is relieved [18,23,24]. In the absence of stress, QC divisions mostly occur periclinally and the resulting daughters are incorporated in the columella lineage, as shown by clonal analyses [18]. The fact that, under physiological conditions, the QC acts mainly as a reservoir for the columella is further substantiated by the observation that in the case of the premature differentiation of CSCs—for instance, in the presence of altered auxin levels—the QC divides to replenish the missing stem cells (Figure 1D) [25]. In addition, the QC plays a key role in maintaining the stemness of the abutting CSCs. Seminal laser ablation experiments demonstrated that the removal of QC cells causes the immediate differentiation of abutting CSCs, suggesting the presence of a QC-derived, non-cell-autonomous signal required for CSCs maintenance [16]. Relevantly, CSCs also may act non-cell-autonomously to protect the QC. At low temperatures, CSCs undergo a single round of division, after which CSCDs die as a consequence of chilling stress. CSCDs’ death maintains QC quiescence and protects the SCN from chilling stress damage through an auxin-regulated mechanism [26]. Thus, it is becoming apparent that QC and CSCs might act as a whole in controlling SCN activity, as further suggested by a number of recent studies [17,20].

## 4. Multiple Regulatory Networks Control DSC Maintenance and QC Quiescence

**Figure 2:**
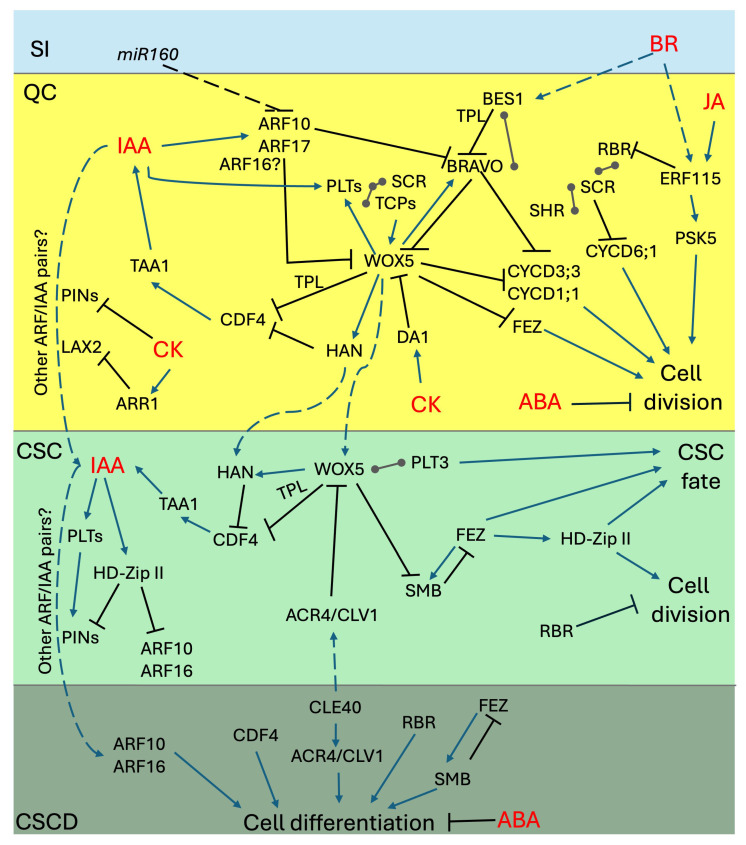
Complex regulation of SCN maintenance. Schematic representation depicting the regulatory links among the main molecular networks governing SCN maintenance and DSC fate. Colored boxes represent specific stem cell compartments to display both cell-autonomous and non-cell-autonomous regulatory steps; see text for details. Blue arrows, positive regulation; black lines, negative regulation; ball-end connectors, protein–protein interactions. Hormone inputs are indicated in red; non-cell-autonomous inputs are depicted with dashed arrows/lines. SI, stele initials; QC, quiescent center; CSC; columella stem cells; CSCD, Columella stem cell daughters.

In recent decades, several research efforts have been made to identify the regulators underlying the activity of root SCN [1,2,27]. One concept that emerged from these studies is that the regulation of DSCs maintenance and QC quiescence cannot be attributed to a single pathway. Rather, there are several independent signals that converge on stem cells as either proliferation- or differentiation-inducing inputs [12,28]. The nature of the signaling molecules involved in the regulation of the SCN activity is quite diverse, as a number of transcription factors (TFs), phytohormones, secreted peptides, and receptors were shown to contribute to the regulation of QC/DSC maintenance [9,27]. While these molecules might primarily act in parallel to regulate cell fate and division in the SCN [12], a number of connections among different inputs have started to emerge, suggesting the existence of high-complexity networks [17,29]. In the following sections, we will review the main regulatory molecules controlling QC quiescence and DSC fate and provide an overview of the relevant connections among different pathways. A simplified graphical representation of relevant connections among the main regulatory networks across stem cell types is presented in Figure 2.

## 5. WOX5: The Master Regulator of DSCs

The observation that CSC maintenance could be regulated by a non-cell-autonomous signal from the QC was further supported by the identification of WUSCHEL-RELATED HOMEOBOX 5 (WOX5), a homeodomain TF specifically expressed in QC cells [30]. Relevantly, genetic ablation of WOX5 causes the simultaneous loss of QC quiescence and the differentiation of CSCs, suggesting both cell- and non-cell-autonomous functions for this TF in the SCN [30].

### 5.1. Cell-Autonomous and Non-Cell-Autonomous Functions of WOX5

In the QC, WOX5 promotes quiescence by repressing the expression of D-type *CYCLIN* (*CYCD*) genes *CYCD1;1* and *CYCD3;3*, suppressing the latter by binding directly to its promoter [31]. In CSCs, WOX5 promotes stemness by repressing the expression of the differentiation-promoting factor CYCLING DOF FACTOR 4 (CDF4) [32]. WOX5 directly binds to the promoter of *CDF4* and silences its expression through an epigenetic mechanism requiring the functions of the TOPLESS (TPL) corepressor and the HISTONE DEACETYLASE 19 (HDAC19) chromatin modifier [32]. Interestingly, this regulatory step requires the translocation of the WOX5 protein from the site of synthesis—the QC—to the site of action—the CSCs—identifying WOX5 as the organizer-derived signal that regulates CSC stemness [16,32]. In agreement with this, the overexpression of WOX5 can revert differentiated columella cells to a CSC-like state [30,32]. It is worth mentioning that the role of WOX5 as an organizer-derived signal is still a matter of debate. In fact, additional evidence indicates that although the translocation of WOX5 from the QC to the CSCs does occur, it might not be strictly required for CSC maintenance, suggesting the presence of additional non-cell-autonomous signals in the QC [28]. Interestingly, one of these signals could be represented by HANABA TARANU (HAN), a GATA TF whose expression is indirectly activated by WOX5 and that, together with WOX5, negatively regulates the expression of *CDF4* in a coherent feed-forward loop [33]. Although these lines of evidence strongly suggest a primary role as a negative regulator of transcription, a recent study investigating the transcriptional and epigenetic landscape of QC cells identified WOX5 as an ambivalent TF capable of both activating and repressing gene expression [34]. In QC cells, WOX5 downregulates a number of cell division-related genes, confirming its role as a master regulator of QC quiescence [34]. At the same time, WOX5 activates, at a very low level, a number of genes specific to mature cells, leading to the hypothesis of a WOX5-mediated priming to confer pluripotency to QC cells in cases of stem cell damage and subsequent regeneration [34]. Interestingly, WUSCHEL (WUS), the WOX5 paralog active in the shoot SCN, also behaves as an ambivalent regulator of gene expression upon the formation of complexes with different TFs [34,35].

### 5.2. WOX5 as a Key Hub in SCN Maintenance

It was recently shown, using Förster Resonance Electron Transfer coupled with Fluorescence Lifetime Imaging Microscopy (FRET-FLIM), that WOX5 is capable of heterodimerizing with members of the PLETHORA (PLT) TFs family: PLT1, PLT2, PLT3, and PLT4 [20]. Furthermore, WOX5 and PLT3 were later shown to also interact with the MYB TF BRASSINOSTEROID AT THE VASCULAR AND ORGANIZING CENTER (BRAVO) [17,36]. Interestingly, the three proteins are capable of forming different heterodimers as well as the heterotrimer, and it has been proposed that the formation of cell-type-specific complexes might be favored by the relative abundance of each protein in a given stem cell compartment [17]. The WOX5-PLT3-BRAVO complex seems to be specific to the QC, whereas WOX5-PLT3 dimers seem to be specific to CSCs [17]. Relevantly, PLT3-WOX5 interactions specifically occur in nuclear bodies and require a PLT3 prion-like domain [20]. It is worth mentioning that the precise regulatory functions of these complexes remain to be established. Mathematical modeling suggested that the BRAVO-WOX5 complex might positively regulate the expression of WOX5 in the stem cells [36]. However, it has been observed that both WOX5 and BRAVO can interact with the TPL corepressor [17,32], suggesting that some of these complexes might act as negative regulators of gene expression. Also, it should be noted that transcriptional cross-regulation among these TFs has been reported [20,25,36]. In summary, while some of its functions have not been uncovered yet, WOX5 represents the master regulator of the SCN. Given its central role in the regulation of QC quiescence and DSC fate, it is not surprising that WOX5 represents a key hub on which several other signals impinge to control SCN maintenance.

## 6. Auxin: The SCN Morphogen

Auxin (Indole-3-acetic acid, IAA) is a prominent hormone regulating multiple aspects in plant development, including the regulation of shoot and root meristematic activities [27,37,38]. In the root SCN, auxin acts as a morphogen by forming an instructive gradient in distal cells, with a hormone maximum in the QC that progressively decreases towards the terminal columella cells [39,40].

### 6.1. The Instructive Role of the Auxin Gradient in the SCN

The distal IAA gradient is established during embryogenesis and further maintained through a combination of hormone biosynthesis and polar auxin transport [41,42,43,44]. In the SCN, IAA is mainly synthesized by TRYPTOPHAN AMINOTRANSFERASE OF ARABIDOPSIS 1 (TAA1), with a minor contribution of TAA1-RELATED 2 (TAR2) [41]. The key role of auxin synthesis in meristem maintenance is clearly demonstrated by the phenotype of the *taa1 tar2* mutant, whose meristematic cells differentiate within a few days after germination [41,43]. Shoot-derived auxin was also shown to be important for the maintenance of the distal IAA gradient [13,40]. On the other hand, a constant reflux loop of IAA within meristematic cells has been shown to help maintain the instructive gradient in distal cells [40,42,45]. Members of the PIN-FORMED (PIN) family of auxin transporters play a key role in coordinating the auxin reflux; in particular, PIN3, PIN4 and PIN7, which are expressed in the SCN, are important for the stabilization of the distal gradient, for SCN patterning, and for columella growth dynamics [25,40,42,45,46]. The instructive IAA gradient controls the balance between proliferation and differentiation in DSCs, maintaining a constant columella root cap size [40]. By following the DR5 auxin transcriptional reporter in columella cells over time, it was shown that CSC division occurs at a relative maximum of DR5 expression, whereas cell separation occurs at a minimum [40]. However, when the distal gradient is disrupted, for instance by increasing auxin levels or by altering its distribution within the SCN, CSCs differentiate and the QC increases its mitotic activity [25]. This response has been linked to an auxin-mediated downregulation of WOX5, which ultimately leads to a *wox5* phenocopy [12,25]. Interestingly, WOX5 also controls IAA biosynthesis through the transcriptional regulation of *TAA1*, suggesting the existence of a negative feedback loop between auxin levels and WOX5-mediated control of SCN [33,47,48]. The mechanisms underlying the auxin-mediated regulation of WOX5 are still unclear and likely involve several components of the nuclear auxin pathway [25,48,49,50]. The transcription factors of the C-class AUXIN RESPONSE FACTOR (ARF) family ARF10 and ARF16 were initially suggested as negative regulators of *WOX5* in response to auxin [25]. Mutants lacking ARF10 and ARF16, as well as plants overexpressing a microRNA (*miR160*) that targets their transcripts for degradation, display an increased number of CSC-like cells in their root cap, with no or a very low presence of mature columella cells [25,51], suggesting their role as repressors of stem cell divisions. However, later genetic evidence suggested that these ARFs act in parallel with, rather than upstream of, WOX5 [12]. More recently, it was shown that *miR160* excludes the transcripts of *ARF10* and of another C-class *ARF17*—but not that of *ARF16*—from the QC [52]. In the absence of *mir160*, ARF10 directly represses *BRAVO* expression by binding to the Auxin-Responsive Elements (AREs) in its promoter [52]. This suggests that *mir160* promotes quiescence by negatively controlling ARF10/17 expression, thus sustaining *BRAVO* and *WOX5* expression in the QC [52]. The role of transcriptional repressors Aux/IAAs in controlling the activity of ARF10 and ARF16 in stem cell identity maintenance is also of particular interest [49]. The canonical, auxin-degradable IAA5 was shown to compete with the non-auxin-degradable IAA33 for the binding of ARF10 and ARF16. This competitive binding was proposed to modulate the activities of repressor C-class ARF10 and ARF16 and that of other activator A-class ARFs in a combinatorial manner to finetune transcriptional responses to changing IAA levels in the SCN [49]. It should be pointed out that, while all the abovementioned studies reported C-class ARFs as transcriptional repressors, a recent work identified transcriptional activation domains in C-class ARFs [53]. It was later shown that ARF10 and ARF16 are required in the SCN for the correct activation of synthetic auxin transcriptional reporters with different *cis*-element architectures compared to DR5 [54]. These data thus suggest a more complex combinatorial action of ARF activities in the SCN, implying that the regulatory roles of C-class ARFs in stem cell fate need to be re-evaluated in light of these new findings. Interestingly, ARF10 and ARF16 are not only subjected to auxin-mediated regulation, as previously reported [51], but they might be part of more complex networks. It was recently shown that three HD-Zip II TFs, namely HAT3, ATHB4, and ATHB2, counteract the roles of ARF10 and ARF16 in auxin-mediated CSC differentiation [29]. ARF10 and ARF16 are mis-expressed in *hat3 athb4 athb2* mutants, and as a result the auxin signaling in the SCN of the triple mutants is altered [29]. Since HD-Zip II TFs display features of transcriptional repressors [55], it is likely that they act to modulate auxin signaling in the SCN, possibly as part of a feedback loop, as at least ATHB2 was shown to be induced by IAA [29]. It is worth mentioning that, in *hat3 athb4 athb2*, the expression of *PIN3* and *PIN4* is also altered, suggesting that these TFs affect multiple aspects of auxin homeostasis in the SCN [29].

### 6.2. The PLT Family in IAA-Mediated SCN Patterning

The PLT family of TFs is also considered a readout of IAA action in the regulation of SCN patterning [25,56]. PLT1, PLT2, PLT3, and PLT4 expression is centered around the SCN, with a graded expression in the proximal meristem that fades towards the transition zone [56,57]. This typical expression pattern follows the graded distribution of IAA within the RAM, although PLT genes are indirect targets of auxin signaling [56,57]. PLTs were shown to act as dose-dependent regulators of stem cell activity: PLTs determine the zonation of meristematic tissues by coordinately activating proliferation genes and repressing differentiation genes [57,58,59]. At the SCN level, it was shown that multiple combinations of *plt* mutants display defects in SCN patterning and CSC maintenance [20,56,57]. While some of the phenotypes observed in higher order *plt* mutants can be determined through an altered distribution of IAA as a result of the defective expression of *PIN1* and *PIN3* [57], most of the SCN activity in PLT TFs is caused by their mutual interactions with WOX5 and other relevant factors. As previously discussed, PLTs are part of the protein complexes comprising WOX5 [17,20,60]. Remarkably, WOX5 and PLTs were shown to regulate each other’s expression in the SCN, with PLTs limiting the *WOX5* expression domain [20,60]. In particular, PLT-mediated regulation of *WOX5* was shown to occur through a high-order transcriptional complex involving SCARECROW (SCR) and Teosinte-branched/Cycloidea/PCNA (TCP) plant-specific TFs [60]. Genetic analyses demonstrated that TCP20, PLT1, PLT3, and SCR act in a dosage-dependent manner to positively regulate *WOX5* expression, to establish a functional SCN during embryogenesis, as well as to maintain post-embryonic QC quiescence and CSC divisions [60]. These data indicate that the establishment of the SCN requires inputs from the longitudinal (PLTs) and the radial (SCR) root patterning plans.

## 7. The FEZ and SOMBRERO Loop: The Pacemaker of CSC Divisions

Another relevant point of control in DSC patterning and maintenance is represented by the cross-regulatory loop between the NAC TFs FEZ and SOMBRERO (SMB) [15]. FEZ and SMB are specifically expressed in the CSC and LRC/epidermis initials to set the pace of coordinated division between the two root cap compartments [12,15]. FEZ promotes formative divisions in the root cap as its genetic mutation reduces the number of CSCs and LRC layers. Conversely, SMB promotes root cap differentiation as an increment in the number of CSC and LRC layers, as can be observed in *smb* mutants [15]. Relevantly, FEZ also plays a role in regulating periclinal cell division planes during wound repair processes [15,61], whereas SMB controls root cap maturation and size [62,63]. In agreement with its role in formative divisions, *FEZ* displays an oscillating expression pattern across CSC/CSCD layers: it is induced in CSCs before the asymmetric cell division, it is partitioned in the two daughter cells upon cytokinesis, and it is finally switched off in the apical cell immediately thereafter [15]. On the other hand, SMB expression is absent in CSCs, and it is expressed in CSCDs immediately after the formative division, suggesting that the two TFs act in a self-regulatory loop. Upon formative division, FEZ has been proposed to induce SMB in CSCDs. In turn, SMB, activates cell differentiation pathways on the one hand, while on the other hand it represses FEZ expression, preventing the further divisions of the CSCDs [12,15]. The positioning of the FEZ/SMB loop across CSC/CSCD layers depends on WOX5 activity [12]. In *wox5* mutants, FEZ is expressed in the organizer position, suggesting that WOX5 prevents QC divisions by actively repressing *FEZ* cell-autonomously. Consistently, SMB expression reaches the CSC layer in *wox5*, likely as a consequence of the upward shift in FEZ expression or of a non-cell-autonomous regulation of WOX5 on *SMB* [12]. Interestingly, FEZ-mediated CSCs proliferation was shown to be dependent on HD-Zip II functions. It was shown that, in *fez* mutants, the expression of ATHB2 and HAT3 in DSCs is abolished. [29]. Intriguingly, ATHB2 displays oscillating expression in CSC/CSCD layers, reminiscent of that of FEZ [29]. Additionally, inducing HAT3 expression in a *fez* background restores CSC proliferation to the wild-type level, suggesting that HD-Zip II acts as an effector of FEZ in CSC maintenance [29]. Remarkably, HD-Zip II TFs connect FEZ-mediated proliferation to IAA-mediated differentiation, suggesting a high degree of coordination between opposing inputs in the regulation of CSC fate [29].

## 8. SHR-SCR-RBR1: Regulators of Asymmetric Cell Divisions

Another relevant point of control of the SCN lies in the interactions among the GRAS TFs SCR and SHORT-ROOT (SHR) and the RETINOBLASTOMA-RELATED protein. SCR and SHR functions are typically associated with the regulation of formative divisions of the cortex/endodermis initials (CEI) by controlling the expression of the *CYCD6;1* gene [64,65,66]. However, *shr* and *scr* mutants display an aberrant SCN patterning and there is evidence indicating a role for these TFs in SCN specification and maintenance [18,67,68]. The plant RETINOBLASTOMA-RELATED (RBR) is the homolog of the human RB1 tumor-suppressor protein and, analogously to its animal counterpart, acts as a key regulator of cell cycle progression [69]. Eukaryotic retinoblastoma proteins exert their role in controlling cell cycle-related transcriptional waves by forming part of large protein complexes: RB-E2F and RB-DREAM [69]. Besides controlling cell cycle progression, plant RBR has been associated with a wide range of plant developmental responses, including DNA damage response, asymmetric cell divisions, and stem cell maintenance [65,69,70,71]. The suppression of the *RBR* transcript, as well as a reduction in RBR protein activity via transgenic phosphovariants, increases the number of divisions in the QC and CSCs, leading to supernumerary stem cells [18,70,72]. Relevantly, upon the silencing of RBR, the QC divides asymmetrically as only the apical daughter cells express WOX5 [18]. While it was suggested that RBR regulates CSC proliferation independently of other pathways, including FEZ/SMB and ARF10/16, the RBR-mediated suppression of asymmetric QC divisions depends on its interaction with the LxCxE motif of SCR [12,18,65]. The enhanced QC divisions in RBR-silenced plants, however, do not depend on CYCD6;1 activity, as observed in CEI [18].

A recent work combining transcriptomics and mathematical modeling proposed that cell-specific stoichiometries of SCR-SHR complexes might determine the capacity of the two TFs to promote divisions in CEI and to repress division in QC [68]. These differences are likely caused by cell-specific differences in the expression of the two TFs, and the work identified SEUSS (SEU) and WOX5 as putative upstream regulators of SHR [68]. Remarkably, SEU was also shown to bind SCR to promote *WOX5* expression via epigenetic regulation [73]. However, it must be pointed out that interactions with other transcriptional regulators might also affect the cell-type specificity of SCR-SHR stoichiometry and, as a consequence, the differential control of stem cell proliferation [74]. A recent work identified TESMIN-LIKE CXC 2 (TCX2) as a putative target of SHR [75]. TCX2 represents a stem-cell-ubiquitous gene expressed across the SCN that has been proposed to regulate the expression of stem-cell-specific gene regulatory networks to coordinate the proliferation of different stem cells types [75]. Relevantly, TCX2 is a putative component of the DREAM complex, and although interactions between RBR and TCX2 have not yet been experimentally demonstrated, it suggests another possible point of contact between RBR and the SCR-SHR network [69,75]. It must also be remembered that the SCR-SHR-RBR network is not isolated from other inputs, as it has been recently shown that the protein BIG, involved in the control of auxin transport, regulates root patterning through a pathway requiring RBR, SHR, and CYCD6;1 [76].

## 9. Peptides and Receptors: Setting the Boundaries Between Stemness and Differentiation

Root and shoot SCNs present an interesting analogy regarding a key regulatory loop that controls stem cell positioning and organization. In the shoot SCN, WUS expression domain defines the OC and, by moving across cell files, WUS specifies the abutting stem cells by promoting the expression of the CLAVATA3 (CLV3) peptide. CLV3, in turn, is secreted outside the cells and restricts the WUS domain by signaling through transmembrane Leucine-rich repeats receptor-like kinases (RLL-RLK) of the CLV1 family [35]. Analogously, in the root SCN, the expression domain of WOX5 was shown to be controlled by a secreted peptide of the CLAVATA3/EMBRYO SURROUNDING REGION (CLE) family, CLE40 [77]. Indeed, CLE40 is expressed in differentiating columella cells and negatively controls CSC proliferation non-cell-autonomously [77]. CLE40 perception occurs via ARABIDOPSIS CRINKLY4 (ACR4) a receptor-like kinase expressed in CSC/CSCD layers, which is induced in the QC upon CLE40 sensing [77,78]. Relevantly, CLV1 expression domain overlaps with ACR4 in CSC/CSCD layers, and it was shown that the two proteins interact and can form heterodimers in planta [78]. Interestingly, ACR4, and to a lesser extent CLV1, are localized in plasmodesmata, and it was suggested that this preferential accumulation helps control the cell-to-cell motility of diffusible factors [78]. Recent evidence, however, indicates that the control of the cell-to-cell motility of stemness-inducing factors by the CLE40-ACR4-CLV1 pathway is unlikely [28]. It was further observed that prolonged treatments with exogenous CLE40 peptide cause an upward shift in the expression of WOX5 and other QC-specific markers in the vascular initials, similarly to what occurs in plants with ablated QC [28]. Interestingly, in proximal cells, CLE40 is not perceived by ACR4/CLV1 as in DSC, but it signals through CLV2/CORYNE (CRN) LRR-RLK receptors [28,79]. Thus, it was proposed that CLE40 signals first promote CSC differentiation by antagonizing QC functions via ACR4/CLV1, then promote the expression of *WOX5* in proximal vascular cells via CLV2/CRN [28]. This feedback signal from differentiating cells might act to re-establish a new SCN once the identity of the original QC is lost upon prolonged CLE40 stimuli, a mechanism that could help maintain the position of the SCN within the RAM in cases of enhanced stem cell differentiation [28].

## 10. Other Factors Involved in SCN Regulation

The regulatory networks discussed above represent the main inputs in SCN maintenance and organization described so far. However, over the years, a number of other inputs—mainly related to hormonal stimuli—have also been identified. Although, in most cases, the regulation of these additional inputs, as well as their connections with the main regulatory networks, remain relatively unexplored, they represent relevant directions for future research in root SCN. A brief overview of the role of these additional inputs is provided below and in Table 1.

### 10.1. Brassinosteroids

Brassinosteroids (BR) have profound effects on cell cycle progression in the RAM and particularly affect DSC dynamics [80]. Exogenous BR applications promote CSC differentiation while enhancing QC divisions, as demonstrated by the mis-expression of QC-specific markers [80]. The BR-mediated enhancement of QC divisions occurs downstream of receptors BR-INSENSITIVE 1 (BRI1), BRI1-LIKE 1 (BRL1), and BRL3, and requires the action of the TFs BR-EMS-SUPPRESSOR 1 (BES1) and BRASSINAZOLE RESISTANT 1 (BZR1) [80,81,82,83]. Interestingly, in SCN cells, BES1 and BZR1 largely display cytoplasmic localization, but upon BR signaling they are translocated into the nuclei, where they regulate the expression of their target genes, promoting QC divisions [24,81]. The QC regulator BRAVO was initially identified among the targets of BES1/BZR1 [24]. Interestingly, BRAVO positively regulates its own expression while being negatively regulated by BES1 in conjunction with the TPL co-repressor [24,84]. Moreso, BRAVO and BES1 form dimers that subtract active monomers to their regulatory functions, thus forming a signaling module that balances the activity of opposing inputs to fine tune QC divisions [24]. As previously discussed, BRAVO regulates QC divisions in conjunction with WOX5 through a complex regulatory mechanism [17,36]. BRAVO heterodimerize with WOX5 and these complexes have been proposed to function through a sequestration mechanism that on the one hand inhibits WOX5 self-repression, and on the other hand confines BRAVO expression to the SCN by limiting the diffusion of a WOX5-dependent mobile signal [36,85]. It is worth mentioning that BR antagonizes the effects of auxin in the regulation of DSC maintenance, suggesting complex interactions between hormonal pathways [81,86].

Another relevant point regarding BR’s role in controlling the SCN lies in the link with stem cell repair mechanisms [23,24,82,87]. It was shown that stem cell regeneration mechanisms in response to genotoxic stress require BR signaling components such as BRL3, BRAVO, and BZR1 to activate restorative QC divisions [24,82,87]. In accordance with their roles in QC divisions, BZR1 is upregulated while BRAVO is downregulated in QC cells upon genotoxic stress [24,87]. Interestingly, the master regeneration competence factor—the AP2/ERF TF ETHYLENE RESPONSE FACTOR 115 (ERF115)—was shown to be induced by BR but not by ethylene [23]. *ERF115* is specifically expressed in QC cells at low levels but it is upregulated in the SCN in response to genotoxic stress in a BRL3-dependent manner [23,87]. ERF115 activates QC divisions in part via the PHYTOSULPHOKINE 5 peptide and in part by sensitizing the wound-surrounding cells to auxin via the class A ARF TF MONOPTEROS [23,88].

### 10.2. Cytokinin

Cytokinin (CK) plays a specific role in controlling the establishment of the SCN during embryo development [89]. By using a synthetic reporter providing a readout of CK transcriptional output, it was shown that CK signaling is strongly active in the hypophysis before the asymmetric cell division that gives rise to the lens-shaped cell (LSC), the QC progenitor [89]. Immediately after division, CK signaling remains high in the LSC, but it is shut down in the basal cell, the progenitor of CSCs, and columella through an auxin-mediated induction of the negative regulators of CK signaling A-type ARABIDOPSIS RESPONSE REGULATOR 7 (ARR7) and ARR15 [89]. The partitioning of high apical CK and high basal IAA signaling ensures the correct embryonic patterning of the SCN [89]. Intriguingly, a similar partitioning of CK and IAA responses was also observed during SCN regeneration in decapitated roots, suggesting that the reactivation of embryonic pathways that lead to hormone signaling separation is a key mechanism for SCN patterning [90]. Interestingly, in post-embryonic development, CK signaling is further switched off in QC cells, regardless of the high local concentration of the hormone [91]. The deactivation of CK signaling in the SCN is required to maintain quiescence, as it was observed that exogenous hormone applications induce QC divisions [19,92]. Similarly, enhancing CK signaling by mutating multiple *A-type ARR* genes induces QC divisions [93]. The CK-mediated promotion of QC divisions has been linked to alterations in distal auxin gradients, as several auxin transporters were shown to be downregulated in high-CK signaling conditions [92,93]. Notably, the promoter of the auxin influx carrier gene *LIKE AUX1 2* (*LAX2*) was shown to be directly bound by ARR1, a CK-responsive B-type transcriptional regulator that is responsible for the repression of *LAX2* [92]. The alterations in distal auxin distribution are not the only effects of the hormone in DSC, as the expression of SCN patterning genes *SCR* and *WOX5* is downregulated by CK [92]. It is not clear whether the downregulation of *WOX5* is a direct effect of the CK action or is mediated by the alterations in the distal auxin gradient. However, WOX5 is also negatively regulated by CK in a post-translational manner, as it was recently shown that the CK-inducible protease DA1 controls WOX5 protein abundance in DSC, suggesting a rather specific action of CK [94].

### 10.3. Ethylene, Abscisic Acid, Jasmonic Acid, and Salicylic Acid

Ethylene (ET), Abscisic acid (ABA), Jasmonic acid (JA), and Salicylic acid (SA), although hormones normally associated with biotic and abiotic stress responses, have all been shown to play a role in controlling SCN maintenance [9,95]. ET was initially shown to promote QC division through the identification of mutants defective in *ETHYLENE OVERPRODUCER 1* (*ETO1*), a gene involved in controlling a key enzyme in the biosynthesis of the ET precursor 1-aminocyclo propane-1-carboxylic acid (ACC) [96]. Interestingly, upon ET-induced divisions, all daughters retain QC identity [96]. While this suggests an increase in the population of QC cells, later analyses demonstrated that ET changes the orientation of cell division planes from anticlinal to periclinal rather than increasing QC proliferation [19]. It must be pointed out that recent evidence for an ET-independent function of ACC in CSC proliferation has been provided [97]; thus, the exact role of these compounds in SCN maintenance remains to be established.

ABA contributes to SCN maintenance by repressing both QC divisions and stem cell differentiation, as demonstrated by experiments with ABA biosynthesis inhibitor fluoridone [98]. Interestingly, fluoridone counteracts the effect of WOX5 overexpression on columella cell dedifferentiation and proliferation, suggesting that the ABA-mediated regulation of SCN impinges on one or more WOX5-dependent pathways [98].

JA plays a relevant role in coordinating SCN maintenance and regeneration by connecting two relevant pathways. It was shown that JA regulates QC quiescence through the SCR-RBR pathway, and at the same time directly promotes the expression of ERF115 [99]. Additionally, RBR and ERF115 interact, linking QC maintenance to SCN regeneration processes [99]. Indeed, the regulatory network downstream of JA is involved in a series of regenerative processes induced through wounding and parasitic infections [99].

Finally, SA was shown to induce QC divisions and concurrently downregulate WOX5 expression. While defects in auxin signaling were observed in plants with high SA levels, it was reported that SA-induced reactive oxygen species (ROS) play a role in controlling SCN maintenance [100]. Relevantly, ROS were also shown to restrict the spatial expression of ERF115 and other ERF TFs, impacting the PSK5 pathway [101].

**Table 1:** Hormone-related molecular players involved in the regulation of the SCN.

Gene	Function	Role in SCN	References
IAA			
*TAA1*, *TAR2*	Tryptophan Amino Transferases	SCN maintenance	[41,43]
*PIN3*, *4*, *7*	Auxin efflux carriers	CSC maintenance	[25,40,42,45,46]
*ARF10*, *16*, *17*	C-class ARF TFs	QC quiescence, CSC maintenance	[49,52]
*IAA5*	Canonical AUX/IAA TF	CSC maintenance	[49]
*IAA33*	Non-Canonical AUX/IAA TF	CSC maintenance	[49]
*HAT3*, *ATHB2, ATHB4*	HD-Zip II TFs	CSC maintenance	[29]
*PLT1*, *2*, *3*, *4*	AP2/ERF TFs	QC quiescence, CSC maintenance	[20,25,56,57]
**CK**			
*AHK2*, *4*	Histidine Receptor Kinase	QC quiescence	[92]
*ARR3-9*, *15*	A-type ARR TFs	SCN establishment, QC quiescence	[89,93]
*ARR1*, *12*	B-type ARR TFs	QC quiescence	[92]
*CKX3*, *5*	CK oxidase/dehydrogenase	QC quiescence	[92]
*LAX2*	Auxin influx carrier	QC quiescence	[92]
*DA1*	Protease	QC quiescence	[94]
**BR**			
*BRI1*, *BRL1*, *BRL3*	LRR Receptor Kinases	QC quiescence	[80,81,82,83]
*BES1*, *BZR1*	BES1/BZR1 TFs	QC quiescence	[80,81,82]
*BRAVO*	R2-R3 MYB TF	QC quiescence	[24]
*ERF115*	AP2/ERF TF	QC quiescence	[23]
**ABA**			
*ABI1*, *2*	Protein Phosphatase 2C	QC quiescence	[98]
*ABI3*	B3 TF	QC quiescence	[98]
*ABI5*	bZIP TF	QC quiescence	[98]
**JA**			
*SHR*, *SCR*	GRAS TFs	QC quiescence	[99]
*RBR*	Cell Cycle Regulator	QC quiescence	[99]
*ERF115*	AP2/ERF TF	QC quiescence	[99]
**SA**			
*NPR1*, *3*, *4*	BTB/POZ–Ankyrin Receptor	QC quiescence	[100]
**ET**			
*ETO1*	Negative Regulator of ACS5	QC quiescence	[96]

IAA, auxin; CK, cytokinin; BR, brassinosteroid; ABA, abscisic acid; JA, jasmonic acid; SA, salicylic acid; ET, ethylene; QC, quiescent center; CSC, columella stem cells; TF, transcription factor; IRR, leucine-rich repeat.

## 11. Concluding Remarks and Future Perspectives

As discussed above, our knowledge of the mechanisms underlying the regulation of the root SCN has made giant steps in recent decades through the identification of key regulators defining properties such as quiescence, stemness, and differentiation in a coordinated manner across different cell layers (Figure 2). The complexity of SCN regulatory networks is quite remarkable and probably several additional layers of regulation are yet to be uncovered. The identification of these additional regulatory steps may be a difficult task, especially considering the limited number of cells constituting the SCN. However, a few relevant questions have surfaced from the data discussed above, and we anticipate that the following four questions may represent key points for future research:

(1) *Cell-to-cell Communication*: While the nature of the QC-derived mobile signal(s) is still under debate, the contribution of symplastic signaling in SCN maintenance has emerged [78,102]. Tools for blocking plasmodesmata are currently available [102,103], and they could be used to better understand how the QC communicates with the stem cells through the exchange of mobile regulators. Relevantly, mobile regulators might not be limited to protein and peptides, as even BR intermediates and microRNAs have been shown to move via plasmodesmata [52,104].
(2) *Complexes’ Complexity*: As we discussed above, several interactions among key SCN regulators have been uncovered, although the exact functions of the resulting multimeric complexes are not yet clear. While some complexes were shown to be transcriptionally active, others were postulated to function through subtracting active monomers from their DNA binding functions [36,74,85]. Also, the exact stoichiometry of the proteins involved within a specific cell type—and possibly their cell-to-cell-movement—might alter the complexes’ composition and activity [17,74]. A precise biochemical characterization of these complexes, along with a combination of FRET-FLIM and fluorescence correlation spectroscopy analyses, will help reveal their functionality, allowing for a fine mapping of SCN’s regulatory circuitry.
(3) *Interpreting the Auxin Readout:* While the instructive role of the auxin gradient is not under debate, most of our knowledge comes from the use of DR5-based transcriptional reporters. In recent years, several lines of evidence have shown that the auxin transcriptional readout is determined by the combinatorial action of activator and repressor ARFs binding to different architectures of ARE pairs in the promoters of auxin-responsive genes [54,105,106]. Considering that many ARFs are expressed in the SCN [107], the current interpretation of DR5-based gradients might be too simplistic. Alternative reporters with different ARE architectures are now available [54], and these tools could help finely dissect the transcriptional readout of IAA gradient in the SCN.
(4) *Unexpected Players: How Many?* While most of the players discussed above are root-specific regulators, in recent years, several TFs previously implicated in other developmental processes were shown to contribute to SCN maintenance. For instance, HD-Zip II TFs were previously implicated in light signaling and shoot development [29,108], NO TRANSMITTING TRACT/WIP-DOMAIN CONTAINING proteins were shown to regulate reproductive development [109], SENSITIVE TO PROTON RIZOTOXICITY 1 was involved in stress responses [110], and C-REPEAT BINDING FACTOR 3 was previously associated with cold acclimation [111]. While some of these factors may play a more general role in regulating hormone homeostasis [112], they may also represent specific signaling hubs that perceive and integrate external cues to synchronize stem cell activity to changing environmental conditions. Understanding how stem cell activity is influenced by the environment will be key to responding to the threats posed by global climate changes.
